# Timeline of diagnosed pain causes in children with severe neurological impairment

**DOI:** 10.3389/fped.2024.1365152

**Published:** 2024-03-06

**Authors:** Francesca Peri, Elena Magni, Filippo Pigani, Raffaella Romoli, Simona Vetrella, Lucia De Zen, Raffaella Sagredini, Egidio Barbi, Giorgio Cozzi

**Affiliations:** ^1^ Department of Medicine, Surgery, and Health Sciences, University of Trieste, Trieste, Italy; ^2^ Department of Pediatrics, Institute for Maternal and Child Health-IRCCS “Burlo Garofolo”, Trieste, Italy; ^3^ Clinical Epidemiology and Public Health Research Unit, Institute for Maternal and Child Health-IRCCS “Burlo Garofolo”, Trieste, Italy; ^4^ Pediatric Unit, Saint Anna Hospital ASST, Como, Italy; ^5^ Department of Primary Assistance—U.O.S.D. Palliative Home-Care, A.S.L. Napoli 1 Centro, Naples, Italy; ^6^ Institute for Maternal and Child Health-IRCCS “Burlo Garofolo”, Trieste, Italy

**Keywords:** pain, pediatric pain, chronic pain, severe neurologic disorder, severe neurologic impairment

## Abstract

**Objective:**

Pain's causes in children with severe cognitive impairment may be challenging to diagnose. This study aimed to investigate if there is a relationship between pain causes and the age of children.

**Methods:**

We conducted a multicenter retrospective study in three Italian Pediatric Units. Eligible subjects were patients from 1 to 18 years with severe neurological impairment. We collected data regarding diagnoses, pain causes and medical or surgical procedures. The timing of pain episodes was categorized into age-related periods: infants and toddlers (0–24 months), preschool children (3–5 years), schoolchildren (6–12 years), and adolescents (13–17 years).

**Results:**

Eighty children with severe neurological impairment were enrolled. The mean age was 11 years (±5.8). Gastroenterological pain was most common in the first years of life (*p* = 0.004), while orthopaedic and tooth pain was the most typical in schoolchildren and adolescents (*p* = 0.001 and *p* = 0.02). Concerning surgical procedures, PEG placement and gastric fundoplication were significantly more common in the first 5 years of age (*p* = 0.03), and heart surgery was typical of infants (*p* = 0.04). Orthopaedic surgery was more commonly reported in older children and adolescents (*p* < 0.001).

**Conclusions:**

Some causes of pain are more frequent in children with severe neurological impairment in defined age-related periods. Specific age-related pain frequencies may help physicians in the diagnostic approach.

## Introduction

Pain in children with severe neurological impairment (SNI) has become a well-defined topic over the last two decades, with literature identifying the leading causes of pain and distress and offering pragmatic assessment tools (1–8). The gained knowledge goes hand in hand with the increased number of children with medical complexity (CMC) and SNI that paediatricians have dealt with in recent years. Various studies offered hints and lists of features that indicate pain in non-verbal children and highlighted possible expressions of pain (1). This assessment is critical in acute pain episodes and in the context of procedural or post-operative pain where there is still a higher risk of undertreatment (9, 10). Gross Motor Function Classification System (GMFCS) is a pragmatic system that further delineates the severity of motor and functional limitations mostly of children with cerebral palsy, but it is also broadly used in other patients with SNI and CMC (11). In addition, caregivers’ role has been prioritized and deemed irreplaceable in reading their children's pain behaviors (12, 13). However, everyday clinical practice reminds us how understanding, diagnosing, and managing pain in these children remains challenging even for appropriately trained operators (14). Thus, children with SNI are still vulnerable to underestimation and undertreatment of pain (15, 16). On a broader perspective, several causes of pain in healthy children are age-related, with a peak of frequency in specific ages of childhood, such as infantile abdominal colic in infancy, earache in toddler and nursery school age, migraine, and functional abdominal pain in adolescents, or with different ages related to the locations of osteochondritis. Therefore, considering the different prevalence of pain causes according to the age of children is a common practice for physicians (17). To our knowledge, no study in the literature investigated whether there is a specific timing for different pain causes in children with SNI, nor for acute, recurrent, chronic, or procedural pain. Knowing that a particular condition may be more likely to occur at a given time could allow a timely diagnosis and an effective and targeted intervention. This study investigates whether painful events could be grouped into specific clusters and the association between the leading causes of pain and age in children with SNI.

## Methods

Our research was a multicenter retrospective study. It was conducted in three urban hospitals in Italy.

- Institute for Maternal and Child Health IRCCS, Burlo Garofolo of Trieste, Trieste
- Pediatric Unit, Saint Anna Hospital ASST, Como, Lariana
- U.O.S.D. Palliative Home-Care, A.S.L., Napoli.

Researchers from the three centers established a study group with experience in dealing with children with SNI and CMC within a pediatric palliative care master course. The coordinating center supervised enrollment and data collection. The Institutional Review Board of the Institute for Maternal and Child Health IRCCS Burlo Garofolo of Trieste, Italy (RC 10/2020) approved the study protocol.

Children with SNI from 0 to 18 years of age, evaluated in the Neurologic Unit, Pediatric Palliative Care Service or Pediatric Ward from January 2023 to June 2023, were considered eligible for participation in the study. We assessed affected by SNI nonverbal children, with GMFCS from IV to V (11), with disorders of the central nervous system characterized by motor and cognitive impairment in the context of medical complexity with the need for medical devices and assistance with daily activities. Neuropsychological evaluation was further integrated into data collection when available.

For every included patient, a team of investigators reviewed the available medical records and collected data, focusing on specific pain episodes, diagnoses, and medical or surgical procedural events to create a patient's timeline of episodes according to age. Data were recorded in a specifically developed electronic database.

Distinctive pain episodes were defined as caused by an identifiable cause based on historical, clinical, lab and imaging clues, responding to a specific treatment, in agreement by two evaluating physicians. They were noted and counted according to the temporal order in which they were detected for the first time. Distinct pain episodes were categorized as follows:

- Gastroenterological pain: constipation, gastroesophageal reflux disease (GERD), post-fundoplication wrenching, pancreatitis, unspecified abdominal pain related to nutrition.
- Orthopaedic pain: hip dislocation, scoliosis, bone fractures
- Neurological pain: neuropathic pain
- Urinary tract infections, renal stones
- Dental caries or abscesses
- Devices complications
- Other: muscular spasms and dystonias.

We recorded surgeries, medical procedures, such as botulinum toxin injections or dental caries treatment, and blood samplings every time we detected them, along with each patient's medical history. The number of hospitalizations for each enrolled patient was recorded, as well.

We categorized the following age-related periods: infants and toddlers (0–24 months), preschool children (3–5 years), schoolchildren (6–12 years), and adolescents (13–17 years).

The primary study aim was to detect if specific pain causes were associated with precise patients’ ages. Secondarily, we aimed to describe the number of times these patients required hospitalizations.

### Statistical analysis

Data were written as numbers and percentages for categorical variables and as mean and standard deviation or as the median and interquartile range (IQR) for continuous variables if normality was unmet. When appropriate, the chi-square or Fisher's exact test assessed differences in the proportions of specific pain episodes and surgeries between age-related periods. The significance level was set equal to a *p*-value of 0.05, and all statistical analysis was carried out using R Software, Version 4.1.1 (R Foundation for Statistical Computing, Vienna).

## Results

During the study period, 85 children with SNI were considered eligible for the study. Five (5.9%) were excluded because their parents did not consent to participation or the medical records review. Finally, 80 children with SNI were enrolled. The mean age was 11 years (±5.8). Fifty-one (63.8%) were male. Among the included children, 27 (33.8%) had a diagnosis of cerebral palsy, 19 (23.8%) of genetic syndromes, 11 (13.7%) of chromosopathies, 9 (11.3%) of epileptic encephalopathies, 4 of brain malformations (cerebropathy, 5.0%). Seven patients (8.7%) presented with more than one condition. Overall, 54 (67.5%) had a history of seizures.

The main clinical and demographic characteristics of the study population are reported in Table 1.

**Table 1:** Clinical characteristics of the population.

Demographic and clinical characteristics	Patients *N* = 80
Age	
Mean (SD)	11 years (±5.8)
Sex, *n* (%)	
Male	51 (63.8)
Female	29 (36.2)
PEG, *n* (%)	
Yes	48 (60.0)
No	31 (38.8)
ND	1 (1.2)
History of seizure *n* (%)	
Yes	54 (67.5)
No	25 (31.3)
ND	1 (1.2)
GMFCS, *n* (%)	
4–5	63 (78.8)
ND	17 (21.2)
Underlying conditions, *n* (%)	
Tetraparesis	27 (33.8)
Syndromes	19 (23.8)
Chromosomopathy	11 (13.8)
Epileptic encefalopathy	9 (11.2)
Multiple conditions	7 (8.7)
Cerebropathy	4 (5.0)
Unknown	3 (3.7)

PEG, percutaneous endoscopy gastrostomy; GMFCS, gross motor function classification system; ND, not determined.

### Specific pain episodes

The population's study registered and reviewed 200 specific pain episodes (Pie Chart 1).

**PIE CHART 1:**
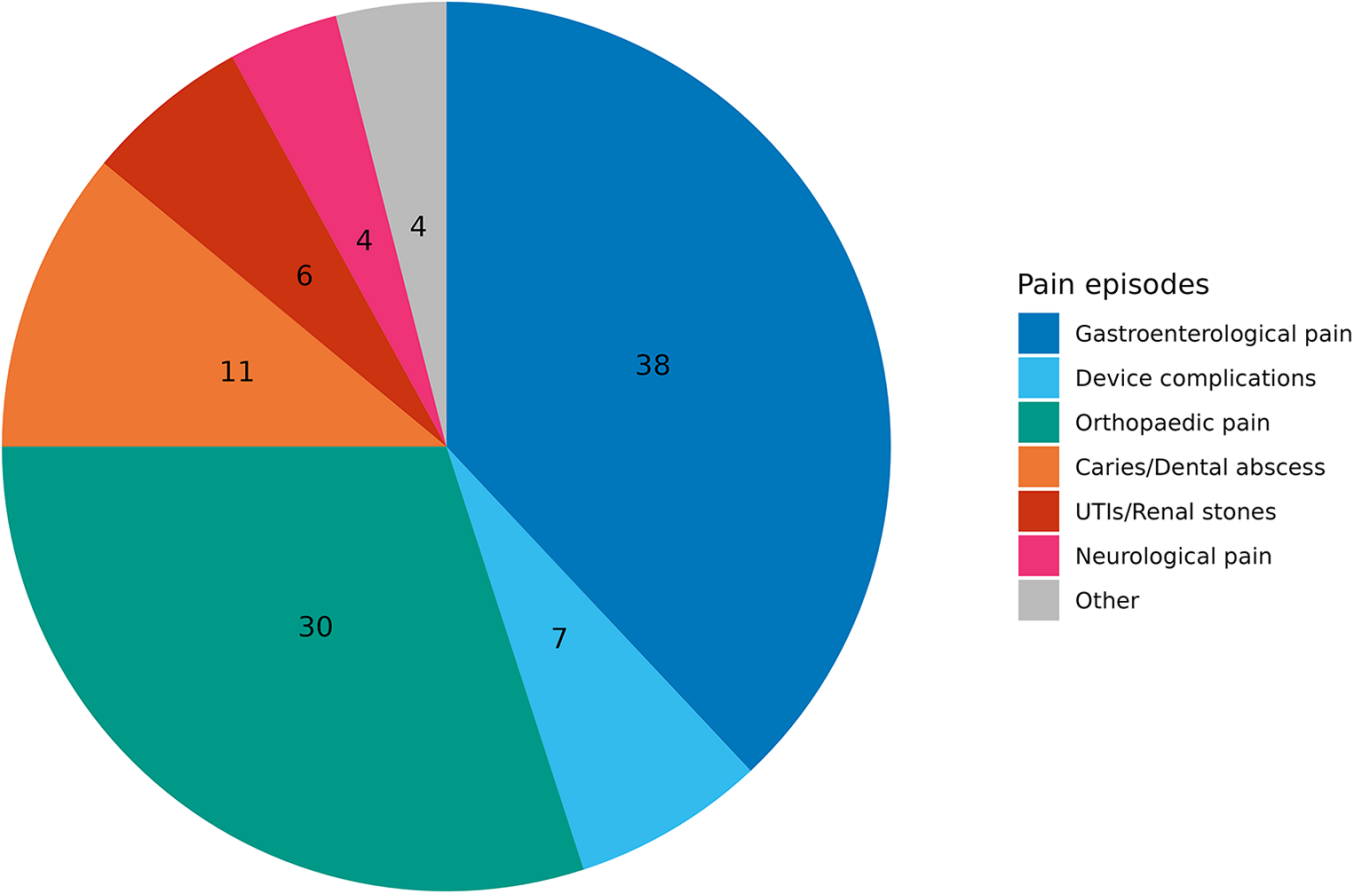
Percentage of total specific pain episodes. Other = dystonia and muscle spasms.

Gastroenterological causes were the most frequently recorded, accounting for 76 (38%) episodes. Sixty (30.0%) pain episodes were related to orthopaedic causes: 28 (13.9%) spine deformities, 20 (9.9%) hip dislocations, and 12 (6.0%) bone fractures. Twenty-two episodes (11.0%) were related to tooth decay. Fourteen episodes (7.0%) were related to abdominal causes that needed surgery, such as inguinal hernias, urinary stones, pancreatitis and cholelithiasis. Fourteen (7.0%) PEG-related device complications were recorded as buried bumper syndrome and peristomal infection. Dystonia and muscle spasms accounted for 4% (see other, Pie Chart 1).

**Figure 1:**
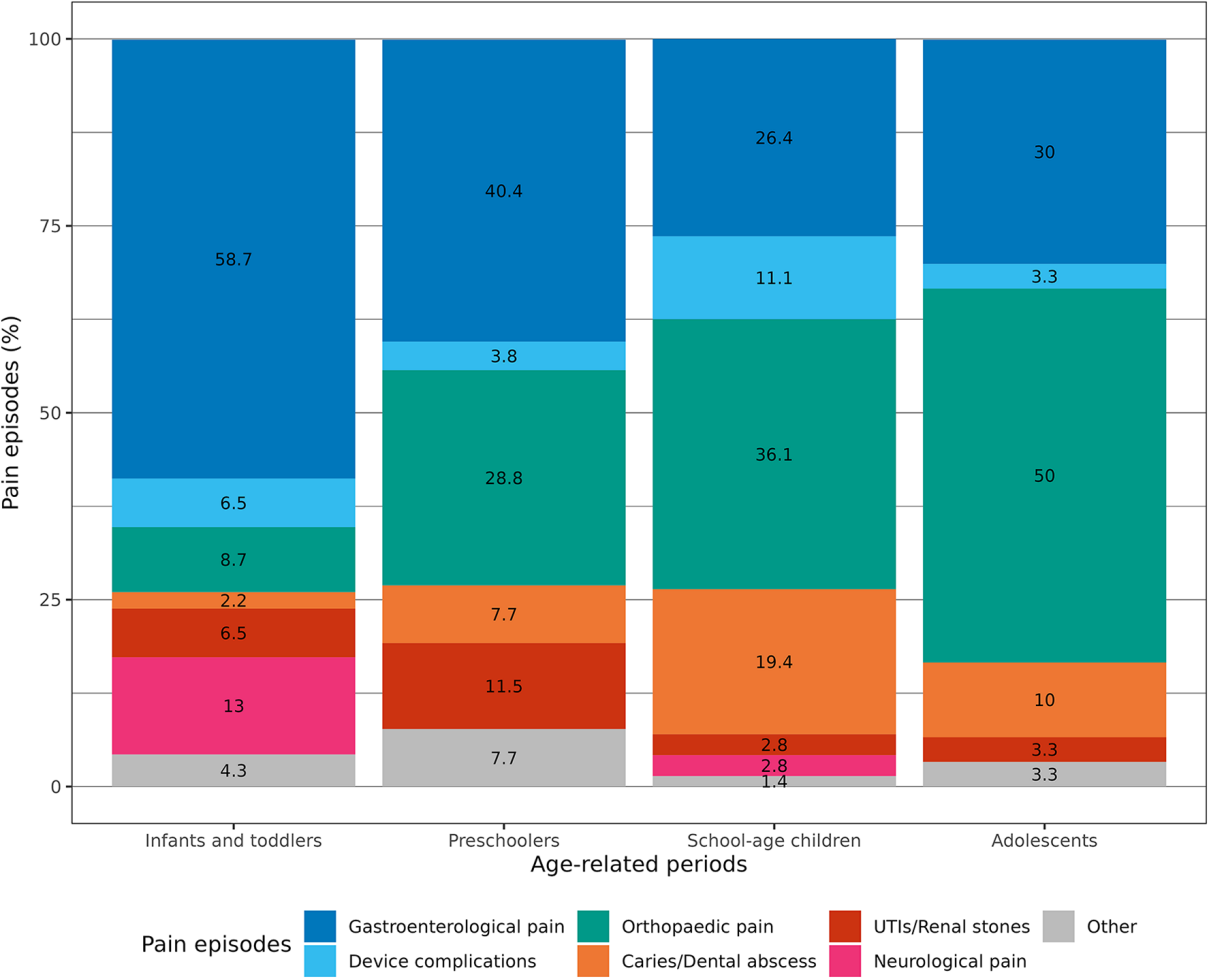
Percentage of age-related specific pain episodes.

Comparing the causes of pain with the age of the patients, we found that gastroenterological pain was significantly more common in the first years of life (*p* = 0.004). On the other hand, orthopaedic pain causes were substantially more common in schoolchildren and adolescents (*p* = 0.001). In the same way, dental caries and abscesses were more commonly reported in children older than 5 years of age (*p* = 0.02). No significant age-related differences existed for device complications and urologic causes (Table 2; Figure 1).

**Table 2:** Age-related specific pain episodes and procedures.

	Age-related periods					
	Total	0–2 y.o.	3–5 y.o.	6–12 y.o.	13–18 y.o.	*p*-value
Specific pain episodes, *n* (%)	200 (100)	46 (23.0)	52 (26.0)	72 (36.0)	30 (15.0)	–
Gastroenterological pain	76 (38.0)	27 (35.5)	21 (27.6)	19 (25.0)	9 (11.9)	**0**.**004**
Device complications	14 (7.0)	3 (21.4)	2 (14.3)	8 (57.2)	1 (7.1)	0.44
Orthopaedic pain	60 (30.0)	4 (6.7)	15 (25.0)	26 (43.3)	15 (25.0)	**0**.**001**
Caries/Dental abscess	22 (11.0)	1 (4.6)	4 (18.2)	14 (63.6)	3 (13.6)	**0**.**02**
UTIs/Renal stones	12 (6.0)	3 (25.0)	6 (50.0)	2 (16.7)	1 (8.3)	0.23
Hydrocephalus	8 (4.0)	6 (75.0)	0	2 (25.0)	0	**0**.**005**
Other	8 (4.0)	2 (25.0)	4 (50.0)	1 (12.5)	1 (12.5)	0.35
Surgery, *n* (%)	162 (100)	40 (24.7)	40 (24.7)	64 (39.5)	18 (11.1)	-
Abdominal	42 (25.9)	11 (26.2)	12 (28.6)	16 (38.1)	3 (7.1)	0.55
PEG	49 (30.3)	16 (32.6)	17 (34.7)	12 (24.5)	4 (8.2)	**0**.**03**
Orthopaedic	18 (11.1)	0	2 (11.1)	9 (50.0)	7 (38.9)	**<0**.**001**
Spasticity	17 (10.5)	2 (11.7)	3 (17.7)	9 (52.9)	3 (17.7)	0.51
Ophthalmic	6 (3.7)	0	1 (16.7)	5 (83.3)	0	0.27
Head and neck	14 (8.6)	1 (7.1)	4 (28.6)	8 (57.2)	1 (7.1)	0.42
Neurosurgery	9 (5.6)	5 (55.6)	0	4 (44.4)	0	0.05
Heart	7 (4.3)	5 (71.4)	1 (14.3)	1 (14.3)	0	**0**.**04**

Bold values indicate significant *p*-values <0.05.

### Procedural pain episodes

One hundred sixty-two surgeries and medical procedures were registered (see Pie Chart 2). Forty-two (25.9%) pain episodes were related to general surgery and 49 (30.3%) to PEG placement. Among general surgery, 19 Nissen fundoplication (10.0%), 13 orchidopexy (7.0%), 6 inguinal hernias repair (3.0%), 3 pyloric dilation (2.0%), one cholecystectomy (1.0%) and one pancreatic surgery (1.0%) were recorded. Orthopedic and spasticity treatments with botulinum toxin injections and baclofen pump placement were performed 18 (11.1%) and 17 (10.5%) times, respectively. Head and neck, neurosurgical procedures, heart surgery and ophthalmic procedures counted for 14 (8.6%), 9 (5.6%), 7 (4.3%) and 6 (3.7%) respectively.

**PIE CHART 2:**
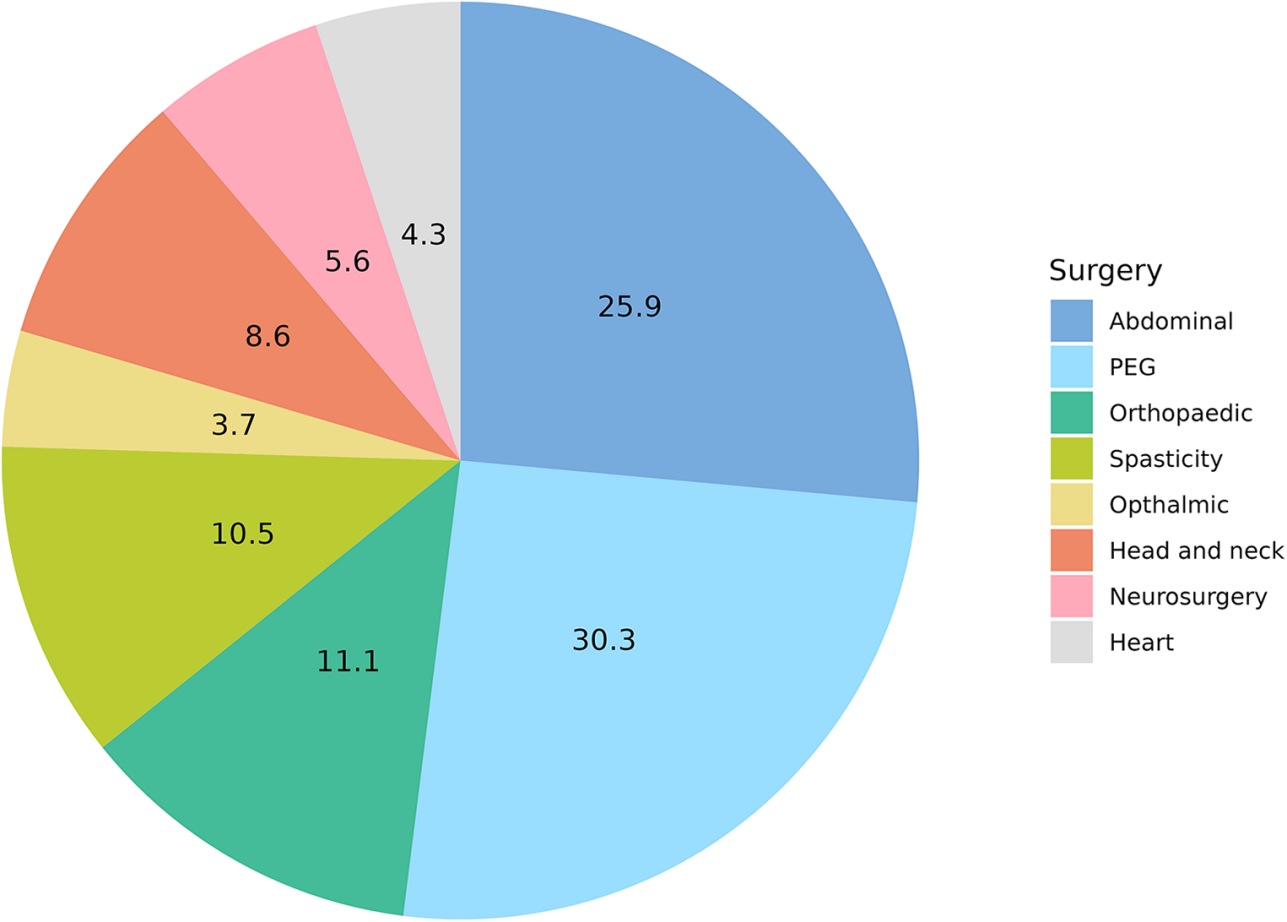
Percentage of total surgeries and medical procedures.

**Figure 2:**
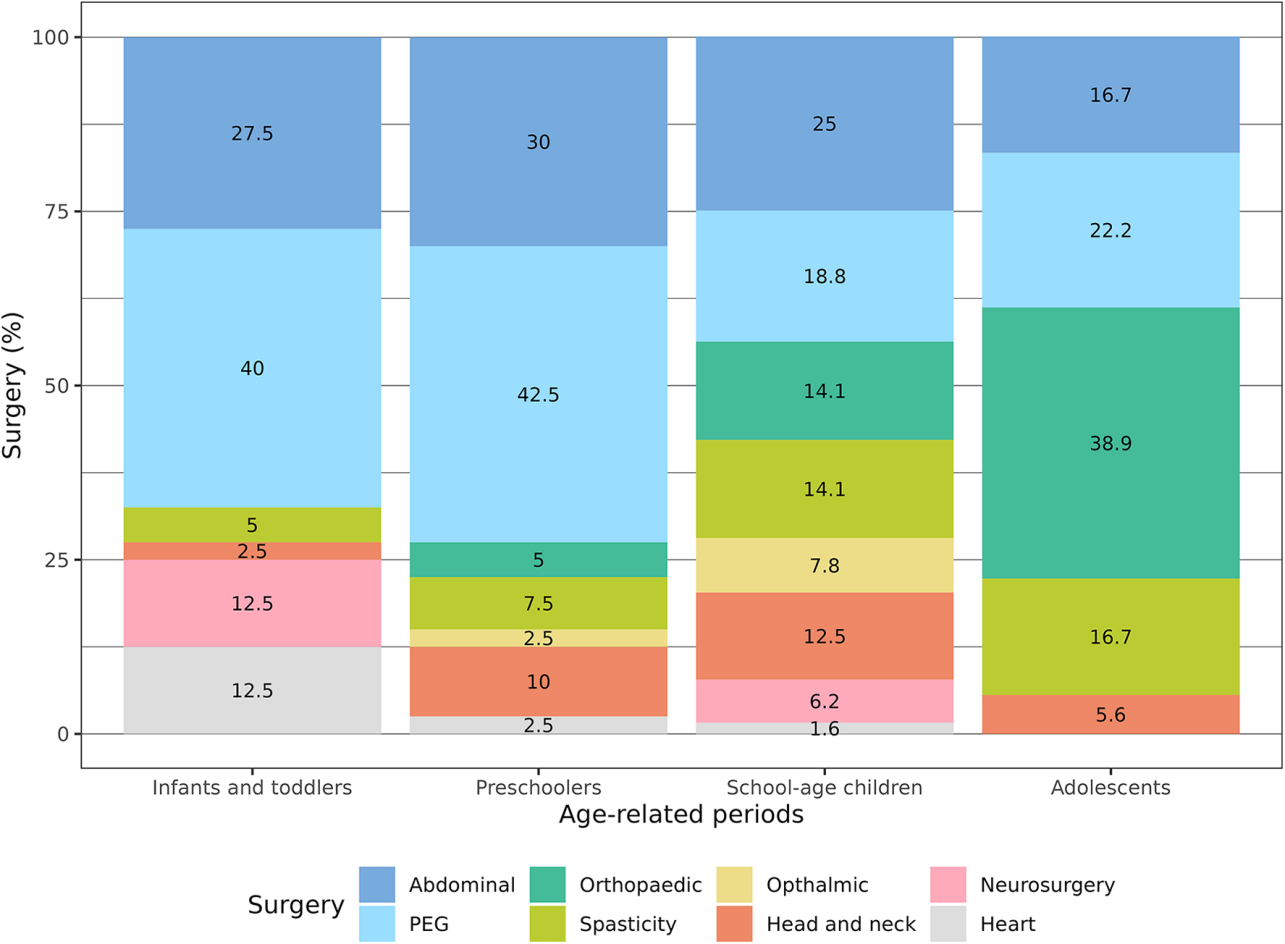
Percentage of age-related surgeries and medical procedures.

Comparing the episodes of surgery and medical procedures with the age of the patients, we found that PEG placement was significantly more common in the first 5 years of age (*p* = 0.03). On the other hand, orthopaedic surgery in terms of bone fractures surgical management and spine surgery was more commonly reported in schoolchildren and adolescents (*p* < 0.001). Heart surgery was recorded mainly in infants (*p* = 0.04) (Table 2; Figure 2).

### Blood samplings

Almost all patients experienced at least one venipuncture in the last year (Table 3), with 19 patients (23.8%) who underwent from 5 to 10 blood tests and 13 patients (16.2%) with more than 10 blood tests. Overall, 53.8% of patients underwent more than 30 venipunctures during their lifetime.

**Table 3:** Overview of blood samplings and hospitalization in our population.

Blood tests and hospitalizations	Patients *n* = 80
Blood tests/year, *n* (%)	
<5	47 (58.8)
5–10	19 (23.8)
>10	13 (16.2)
ND	1 (1.2)
Blood tests/life, *n* (%)	
<10	5 (6.2)
10–30	32 (40.0)
>30	43 (53.8)
Hosp. admissions/year, *n* (%)	
0	13 (16.2)
1–2	30 (37.5)
3 or more	18 (22.5)
ND	19 (23.8)
Hosp. admissions/life, *n* (%)	
<5	25 (31.3)
5–10	25 (31.3)
>10	11 (13.7)
ND	19 (23.7)
Length of stay/life, *n* (%)	
<4 weeks	19 (23.8)
4–12 weeks	21 (26.2)
12–40 weeks	22 (27.5)
>40 weeks	8 (10.0)
ND	10 (12.5)

## Hospitalization rate

Thirty (37%) children were hospitalized at least one time over the last year, and 18 children (22%) three or more times (Table 3). On the contrary, 13 patients (16%) were not admitted. Eleven patients (14%) visited the hospital more than ten times during their lives, 25 subjects (31%) were admitted between five and ten times and 25 (31%) were admitted less than five times.

In this population, most children were hospitalized for more than one month, with 22 patients (27.5%) hospitalized between 12 and 40 weeks and 8 patients hospitalized longer than 40 weeks (Table 3).

## Discussion

This study aims at describing the temporal association between painful events and age in pediatric patients with SNI. One of the most significant challenges for paediatricians is the early and correct assessment of pain triggers in non-verbal patients alongside an effective pain relief treatment. Our research confirms well-known literature data about the leading causes of pain in these children (4, 5). Moreover, these results suggest that specific pain episodes may be grouped in specific cluster considering the age of patients. Gastrointestinal pain prevails over other sources during the first years of life. To our knowledge, while our data confirmed the high incidence of GERD in this population (4, 5, 18–21), no previous study highlighted the early onset.

On the other hand, we noted that orthopaedic pain is typical of older children. Hip subluxations, spine deformities and fractures represent a prevalent source of pain in children between 6 and 17 years. The high incidence of hip subluxation and dislocation in these patients is well known, but no age correlation has been previously described. It is well known that children with SNI are prone to fracturing due to several factors such as immobilization, anti-seizure medications, limited exposure to the sun, and poor intake of vitamins and minerals (22–25). Early bone densitometry or preventive bisphosphonates treatment should be offered. The available evidence is still controversial, and its quality must improve (26). Moreover, no study evaluated changes in bone pain or quality of life following bisphosphonates treatment, which, in our experience, is the treatment of choice. Further prospective and well-designed randomized control studies should be performed to address this controversy and define the best timing and duration of treatment.

Common factors in children with SNI such as incomplete bladder emptying, immobility, repeated catheterizations, poor fluid intake, anti-epileptic treatment can lead to repeated urinary tract infections that can further result in struvite stones development (27, 28). Most of the pain episodes in our research has been detected in the first five years probably due to the easy accessibility to urine dipstick and abdomen ultrasound, a reminder that these diagnostic tools are an essential part of the diagnostic work-up (1, 2).

Dystonia and muscle spasms may be interrelated with orthopaedic or “musculoskeletal” pain; for instance, they can be secondary to hip subluxation. However, we believe these are troublesome symptoms and should be considered independently. Recent work highlights the need to address the evaluation and management of dystonia as one of the most challenging symptoms in children with CMC and SNI (29). It is acknowledged that dystonia and spasticity affect many of these patients (2–5), even though they are likely underestimated (30). Dystonia and muscle spasms were possible to be under-identified in our case series as this information is only rarely reported in medical charts.

This study also reminds us that the relevance of iatrogenic pain and distress secondary to blood samplings and hospitalizations represent a significant burden and should not be underestimated. The number of venipunctures in these series is remarkable, and more than half of our population study underwent more than thirty blood samplings over their lifetime. Venipuncture is a distressing procedure in children with SNI compared to healthy peers (31, 32), and these data highlighted the need for pain management strategies in this population, such as the mandatory use of topical anaesthesia. Likewise, the literature highlights the risk of undertreatment of pain after surgery in children with SNI (9, 10). This series’ high number of surgical procedures reminds paediatricians of the need for careful pain evaluation and treatment after surgery. Lastly, this study shows that ten per cent of the population (with a mean age of 11 years) spent more than 40 weeks hospitalized. Balance disruption from acute illnesses or hospitalizations is stressful for both the child and the caregiver (33, 34).

This study has several limitations, mainly secondary to its retrospective design. First, our research underestimated the pain episodes’ precise frequency for several reasons. Even the most accurate physician may fail to detect and report specific pain episodes; medical records do not always match real-life. Moreover, every hospital had its clinical chart with a different data collection style. Different or missing information can lead to bias in both cases. We focused on detecting pain episodes rather than pain assessment with dedicated tools, which could be a further limitation. Unlike pain assessment scales for children without cognitive impairment, whose main strength is that they are based on self-assessment (35–37), pain evaluation in patients with SNI can be only delegated. The literature only partially describes the lack of specific scales in this population. However it may also cause an under-detection of pain (38). Likewise, we were defective in analyzing non-identified or missed pain causes, a issue in this population often with dramatic outcomes (39). Furthermore, our detection method may be debatable. For some long-lasting or recurrent painful conditions such as constipation, scoliosis, and dystonia, we focused on their incidence in terms of the first time they were noted rather than following their course in the medical history of the single patient over the years. Another related limitation was the precise age at which some painful events occurred. Some conditions may have been unrecognized several times or were first detected and treated by family doctors and only reported later in the medical chart. Lastly, we did not report other standard pediatric sources of pain, such as otitis media, menstrual pain, or primary headaches, which were still present in children with SNI, nor did we record any treatment related to chronic pain management. This study also presented some points of strength. It is the first attempt to define the history of painful events in children with CMC and SNI, as we aimed to offer a cursory yet valid overview. Moreover, the multicenter design enriched the heterogeneous but well-balanced population and offered many patients. This retrospective research may set the ground for future prospective and pragmatic studies considering drug use and preventive and therapeutic strategies (see Supplementary Figure S1).

## Data Availability

The original contributions presented in the study are included in the article/**Supplementary Material**. Requests to access these datasets should be directed to francesca.peri@burlo.trieste.it.

## Supplementary material

Supplementary Figure S1 Future research perspective in children with SNI. SNI, sever neurological impairment.
