# Supplementary figures and images for: Timeline of diagnosed pain causes in children with severe neurological impairment
Source: Front Pediatr. 2024 Mar 6;12:1365152. doi: 10.3389/fped.2024.1365152 (PMC10950906; doi:10.3389/fped.2024.1365152)

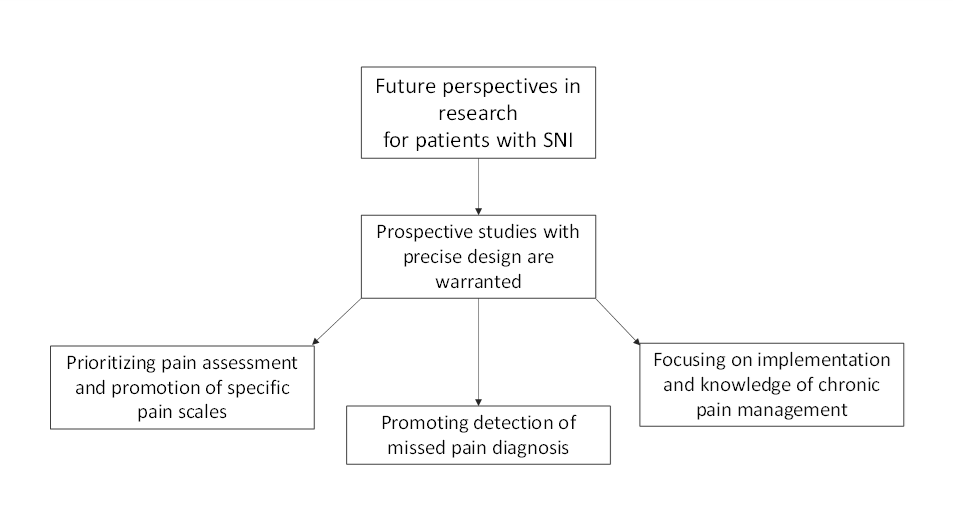

Supplement: Supplementary Figure S1 — Future research perspective in children with SNI. SNI, sever neurological impairment. [file Image1.tif]
